# Case of an Extra-Uterine Fœtus

**Published:** 1785

**Authors:** 


V. *Cafe of an extra-uterine fœtus. Communicated in a letter to Dr.* Simmons, *by Mr.* Richard Moyle, *Surgeon at Marazion, in Cornwall.*

ALSEY Penmear, a poor woman belonging to a neighbouring parifh, after being pregnant three years, difcovered a large tumor about the navel, which, on her jumping over a hedge, burft open, and continued to difcharge, at times, for twelve months (without any material injury to her health), the bones of a half-grown fœtus, after which the wound foon completely healed, and the patient continued to enjoy a good ftate of health.

In the month of January, 1779 (being near three years after the healing of the above wound) a tumour again appeared in the fame part of the abdomen, and gradually increafed till till February 28th, when, in confequence of fome exertion, it burft, and about a yard of inteftine came out through a very fmall aperture. —A few days after this I vifited her, and found the inteftine greatly inflated, grown dry, and in a gangrenous ftate, owing to the violent ftrangulation at the wound.—From the fymptoms which attended, fuch as a conftant vomiting of fœces, hiccup, fhiverings, and a weak fluttering pulfe, I expected that a very fhort time would put an end to her miferable exiftence. But in this I was greatly miftaken, as fhe furvived twelve days after I faw her, and nature was fufficiently ftrong to throw off the gangrenous piece of inteftine clofe to the wound, through which the fæces were difcharged a day or two before her death.

*Marazion,*

*Feb.* 19, 1785.

